# Diagnosis of Achondroplasia at Birth: A Case Report

**DOI:** 10.31729/jnma.4846

**Published:** 2020-02-29

**Authors:** Suzit Bhusal, Uttara Gautam, Rajan Phuyal, Robin Choudhary, Sunil Raja Manandhar, Aliska Niroula

**Affiliations:** 1 Kathamandu Medical College and Teaching Hospital, Sinamangal, Kathmandu, Nepal; 2 Department of Pediatrics Kathmandu Medical College and Teaching Hospital, Sinamangal, Kathmandu, Nepal

**Keywords:** *achondroplasia*, *dwarfism*, *ultrasonography*

## Abstract

Autosomal dominant mutations in fibroblast growth factor receptor 3 cause achondroplasia, the most common form of dwarfism in humans. Achondroplasia is a genetic disorder causing rhizomelic shortening of limbs. Head is often large with prominent forehead causing vaginal delivery difficult. A twenty-one years old multipara mother gave birth to a baby with achondroplasia via spontaneous vaginal delivery with episiotomy without any complication. Achondroplasia, in this case, was diagnosed on the basis of antenatal ultrasonography finding, clinical features and radiological finding of the baby. He was admitted in the special baby care unit for observation and discharged on the next day as no complications were noted.

## INTRODUCTION

Achondroplasia is a nonlethal form of chondrodysplasia and is the most prevalent form of skeletal dysplasia with characteristic short limb dwarfism. It may be inherited by autosomal dominant gene; however, most cases appear as spontaneous mutations. Radiographs of the skull, spine, pelvis, and extremities reveal the characteristic features. Affected individuals have rhizomelic shortening of limbs. Phenotypic features include disproportionate short stature, megalencephaly, a prominent forehead (frontal bossing), midface hypoplasia, rhizomelic shortening of the arms and legs, a normal trunk length, prominent lumbar lordosis, genu varum, and a trident hand configuration.^1^

Although achondroplasia is a well-documented cause of disproportionate short stature, it is difficult to diagnose at birth as compared to children and adults.^2^ People with achondroplasia are generally of normal intelligence. Health problems commonly associated with achondroplasia include episodes in which breathing slows or stops for short periods (apnea), obesity, and recurrent ear infections. In childhood, individuals with the condition usually develop a pronounced and permanent sway of the lower back (lordosis) and bowed legs. Some affected people also develop abnormal front-to-back curvature of the spine (kyphosis) and back pain. A potentially serious complication of achondroplasia is spinal stenosis.^3^ Here we report a case of achondroplasia diagnosed at 1^st^ day of life-based on clinical and radiological features.

## CASE REPORT

The case is of a term male baby delivered at 40 weeks and four days of gestation to a gravida 2, parity 1 (G2P1) with no consanguinity, residing at Arubari, Kathmandu. Baby was born via spontaneous vaginal delivery with mediolateral episiotomy having an APGAR score of 8/10 and 9/10 at 1 and 5 minutes respectively and birth weight of 3.5kg. There was no obstructed labor. Placenta and membrane were completely evacuated with placental weight of 500gm. The estimated blood loss at the time of delivery was 100ml and the post-delivery vitals of mother was stable. Her first baby had normal physical features with the achievement of an appropriate milestone.

Anomaly scan at 22 weeks and 1 day of gestation showed no gross anomaly of the fetus. However, third-trimester scan at 36 weeks and 4 days of gestation from last menstrual period showed a single, live, intrauterine pregnancy corresponding to 35 weeks 1 day of gestation with anterior fundal placenta and cephalic presentation of the fetus with adequate liquor. It also noted dysplastic bilateral short femur and humerus for age but the mother wanted to try a normal vaginal delivery Both parents had achieved average height. There was no family history of rhizomelic shortening of limbs.

**Figure 1:**
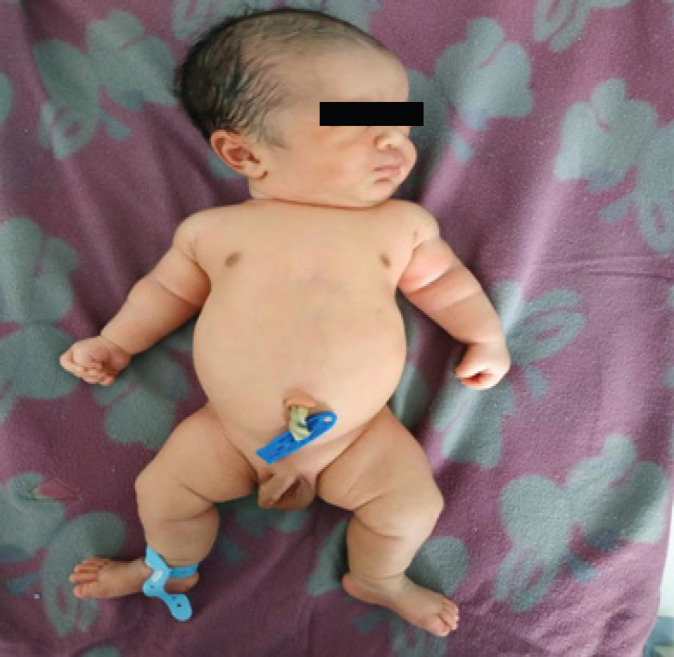
Disproportionate shortening of long bones, large head with frontal bossing, flattening of the nasal bridge and protuberant abdomen.

Baby cried immediately after birth and no respiratory difficulty was noted. On anthropometric measurement, head circumference was 36cm, chest circumference was 32cm, abdominal girth was 30.5cm and length of baby was 46cm. The upper segment was 30.5cm and the lower segment was 15.5cm making an upper segment to lower segment ratio of 1.97. On clinical examination, the baby had a large head with frontal bossing. There was bilateral symmetrical shortening of upper and lower limbs with short fingers. Depressed nasal bridge was present. Abdomen was protuberant. The tone of the baby was normal (Figure 1).

**Figure 2:**
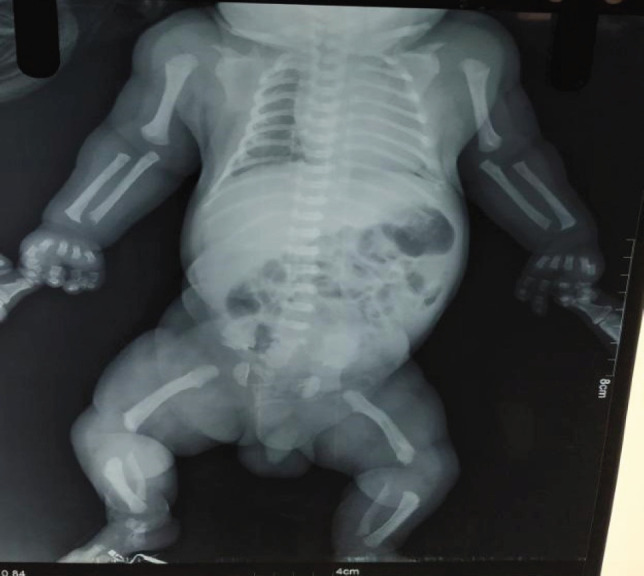
X-ray infantogram showing bilateral humerus and femur shortening.

Baby was taken to the special baby care unit for observation. X-ray was ordered which pointed towards a diagnosis of achondroplasia. It showed a widening of both proximal and distal metaphysis of bilateral humerus and femur suggestive of metaphyseal faring. Bilateral humerus and femur were shortened (rhizomelic shortening). Metacarpals of both hands were short and of similar length with separation of middle and ring fingers (trident hand) (Figure 2).

Baby was observed for 24 hours for any complication and was discharged after counseling the parent.

## DISCUSSION

Achondroplasia is caused by a mutation in the fibroblast growth factor receptor-3 gene (FGFR3), which is located at 4p16.3. It may be inherited as an autosomal dominant trait, which means that if one parent has achondroplasia, the infant has a 50% chance of inheriting the disorder and if both parents have the condition, the infant's chances of being affected increase to 75%. However, most cases appear as spontaneous mutations which means that an achondroplasia child can have normal parents. In our case both parents had achieved average height. The diagnosis is mainly based on clinical and radiological features in the developing world.^3,4^ Achondroplasia has a global incidence of 1/77,000-1/15,000.^5^

It is usually suspected during ultrasound because of shortened long bones.^5^ In our case, third-trimester ultrasound was suggestive of dysplastic short femur and humerus.^6^ It can also be diagnosed on the basis of characteristic clinical and radiographic findings in most affected individuals.^7^ Though diagnosis at birth is relatively difficult as compared to children and adults, our case was diagnosed at birth due to suggestive ultrasound, radiological evidence and distinct clinical features. Clinically patient has a disproportionate shortening of long bones, large head with frontal bossing, flattening of the nasal bridge and protuberant abdomen. Since features of achondroplasia are very characteristic a careful observation would lead to diagnoses of this condition. Due to the socio-economic limitation of the patient genetic testing for FGFR3 couldn’t be done.

Vaginal delivery of a baby with achondroplasia can be complicated due to the large head. In our case, vaginal delivery of the baby was uncomplicated with no significant blood loss.

The risk of recurrence in family with sporadic cases is estimated to be 1 in 443.^8^ This is said to be due to mosaicism in one of the parents. If one of the parents is affected with Achondroplasia the risk to offspring of recurrence is 50% for either sex. If both parents are affected then 25% of children will be normal, 50% heterozygous and 25% will be homozygous mutation. Homozygous Achondroplasia is always lethal.^9^

**Consent:** JNMA Case Report Consent Form was signed by the patient and the original article is attached with the patient’s chart.

## Conflicts of Interest:

None.
